# The Influence of Dementia Caregiving Styles on Caregiver Distress and the Person With Dementia’s Quality of Life

**DOI:** 10.1093/geroni/igaa057.1160

**Published:** 2020-12-16

**Authors:** Amanda Leggett, Cathleen Connell, Laura Gitlin, Helen Kales

**Affiliations:** 1 The University of Michigan, Ypsilanti, Michigan, United States; 2 University of Michigan, Ann Arbor, Michigan, United States; 3 Drexel University, Philadelphia, Pennsylvania, United States; 4 UC Davis, Sacramento, California, United States

## Abstract

Building vertically upon the Stress Process Model, dementia caregivers’ cognitive-behavioral management styles are an understudied area with implications for dyadic care outcomes and tailoring of care interventions. We consider whether membership in five previously classified caregiving styles (Externalizers, Individualists, Learners, Adapters, Nurturers- which vary in their adaptability, dementia understanding, and behavioral management practices) impacts caregivers’ experiences of care-related stress and the quality of life of the person with dementia (PWD). Participants included 100 primary family caregivers for PWDs who were 74% female, 18% non-White, and on average 64 years old. Utilizing linear regressions, each caregiving style was considered as a key predictor (reference: Externalizers- poor understanding, non-adaptable approach, and punitive behavioral strategies) of the Caregiver Assessment of Function and Upset (CAFU) upset score, Neuropsychiatric Inventory (NPI-C) distress scale, Zarit Burden Interview (ZBI), and PWD quality of life (QOL-AD) scale controlling for demographics, care duration, co-residency, and dementia severity. Relative to Externalizers, Nurturers (understanding, adaptability, positive engagements) had less CAFU upset (β=-0.4, p<.01), less NPI-C distress (β=-0.3, p<.05), and greater QOL-AD for the PWD (β=0.4, p<.01). Learners (recognize need to change care approach, attempting adaptability, trial-and-error behavioral care) also showed significantly lower NPI-C distress than Externalizers (β=-0.5, p<.01). Thus caregiving styles with more dementia understanding, adaptability and positive behavioral strategies showed less distress and better PWD QOL. Corresponding with recent dementia care summits calling for identification of caregivers at greatest risk for poor outcomes, targeting and tailoring interventions based on caregiving styles may lead to great public health impact.

